# Dislodgement forces and cost effectiveness of prehospital securement of peripheral intravenous catheters on moist skin: a randomized controlled clinical trial in healthy volunteers

**DOI:** 10.1007/s00068-026-03227-z

**Published:** 2026-06-03

**Authors:** Johannes Vogeler, Stefan Schumann, Sebastian Heinrich, Johannes Hell, Axel Schmutz

**Affiliations:** Medical Center - University of Freiburg, Department of Anesthesiology and Critical Care, Faculty of Medicine, University of Freiburg, Hugstetter Strasse 55, 79106 Freiburg, Germany

**Keywords:** Catheter, Fixation, Dislodgement, Prehospital, Intravenous

## Abstract

**Objectives:**

Peripheral intravenous catheters (PIVC) are widely used clinical devices for administering fluids or essential drugs to patients. The failure rate of PIVC remains high and Emergency Medicine has been shown to be a risk factor for dislodgement. When the skin is moist from sweat or fluids, standard intra-hospital dressings and securements fail. In emergency situations, a failed catheter can then critically delay intravenous therapies. The most effective dressing to prevent accidental removal of a prehospital PIVC remains unclear. It was the aim of this study to compare the force required to dislodge a PIVC with four different methods of securing PIVCs used in emergency medicine. In addition, the costs were calculated.

**Methods:**

Artificial sweat was applied to the skin of 180 volunteers. PIVCs were attached onto the forearm using four different securements (elastic gauze, cohesive gauze, clingfilm and a velcro securing device). Continuously increasing traction force was applied until dislodgement of the respective securement. For statistical tests, either Friedman’s test or repeated measures ANOVA was used.

**Results:**

Clingfilm showed the greatest resistance to increasing pulling force with cohesive bandages as close second. The velcro securing device was strongest at resisting low level of forces but fell off sharply at higher force. Elastic bandages were the weakest in both categories. Clingfilm was the most cost-effective method (4ct), followed by elastic gauze (12 ct), cohesive gauze (60 ct) and the velcro device (316 ct).

**Conclusions:**

In situations where intravenous catheters are difficult to secure or at high risk of dislodgement either clingfilm or cohesive dressing should be used. Simple elastic bandages should not be used in any setting for securement of PIVC.

## Introduction

Prehospital placement of peripheral intravenous catheters (PIVC) is a common invasive procedure routinely performed during emergency medical service. PIVC are an essential component for many lifesaving measures including delivery of medication and fluid resuscitation. Prehospital insertion rates of 20% to 58% are reported, with success rates of 87–94% [1]. However, the skin of emergency patients is often affected by diaphoresis, moisture, blood or soiling and the dressings and securements usually applied in-hospital lack sufficient adhesiveness in the prehospital setting. As PIVC insertion by paramedics is associated with an increased risk of dislodgement, an increased emphasis on securement in the prehospital setting is suggested [2]. PIVC dislodgement puts patients at risk of delay in intravenous therapy and is reported to be as high as 24% [3].

Moreover, a secure dressing limits small movement related irritation inside the vein with related mechanical trauma to the endothelium, leading to thrombophlebitis and eventually device failure [4–6].

There are no guidelines that address the fixation method with regard to the prevention of catheter dislodgement in the prehospital setting and optimal PIVC dressing and securement is still considered unresolved [7]. In the most recent Cochrane systematic review, transparent dressings, bordered and non-bordered, were more effective in preventing dislodgement or accidental removal than a fixation with gauze or a securement device in in-hospital patients [8]. A study carried out by military paramedics in Israel evaluated different prehospital fixations under wet and dry circumstances and reported a resistance to shear force of around 70 N to 100 N [9].

Nevertheless, there is limited data on the effectiveness of fixation methods for prehospital PIVC in terms of dislodgement force. The most effective dressing and securement method to prevent accidental removal of prehospital applied PIVC where standard adhesive dressings fail remains unclear.

We hypothesized that the force required to dislodge a PIVC on moist skin depends on the respective securement method.

For that purpose, we fixated truncated PIVCs with four types of securement at the artificially moistened arms of volunteers and measured the force required to dislodge the PIVC.

As a secondary outcome, we evaluated the resistance against small movements for each of the securements. Additionally, the respective costs of the four investigated securement methods were calculated.

## Methods

### Study design and setting

In this randomized controlled trial, a convenience sample of volunteers was enrolled in an academic surgical department from April 2023 to October 2023. Institutional Review Board approval was provided by the local Ethics Committee (March 23rdth 2023 Approval Number EK 23-1064-S1). The study was registered in the German Clinical Trials Register (DRKS00033875).

### Selection of participants

After written informed consent, a total of 180 healthy volunteers from the hospital´s medical staff were enrolled in the study. Exclusion criteria were non-intact skin on forearm or antecubital region, steroid medication and age < 18 years.

### Interventions

**Fig. 1:**
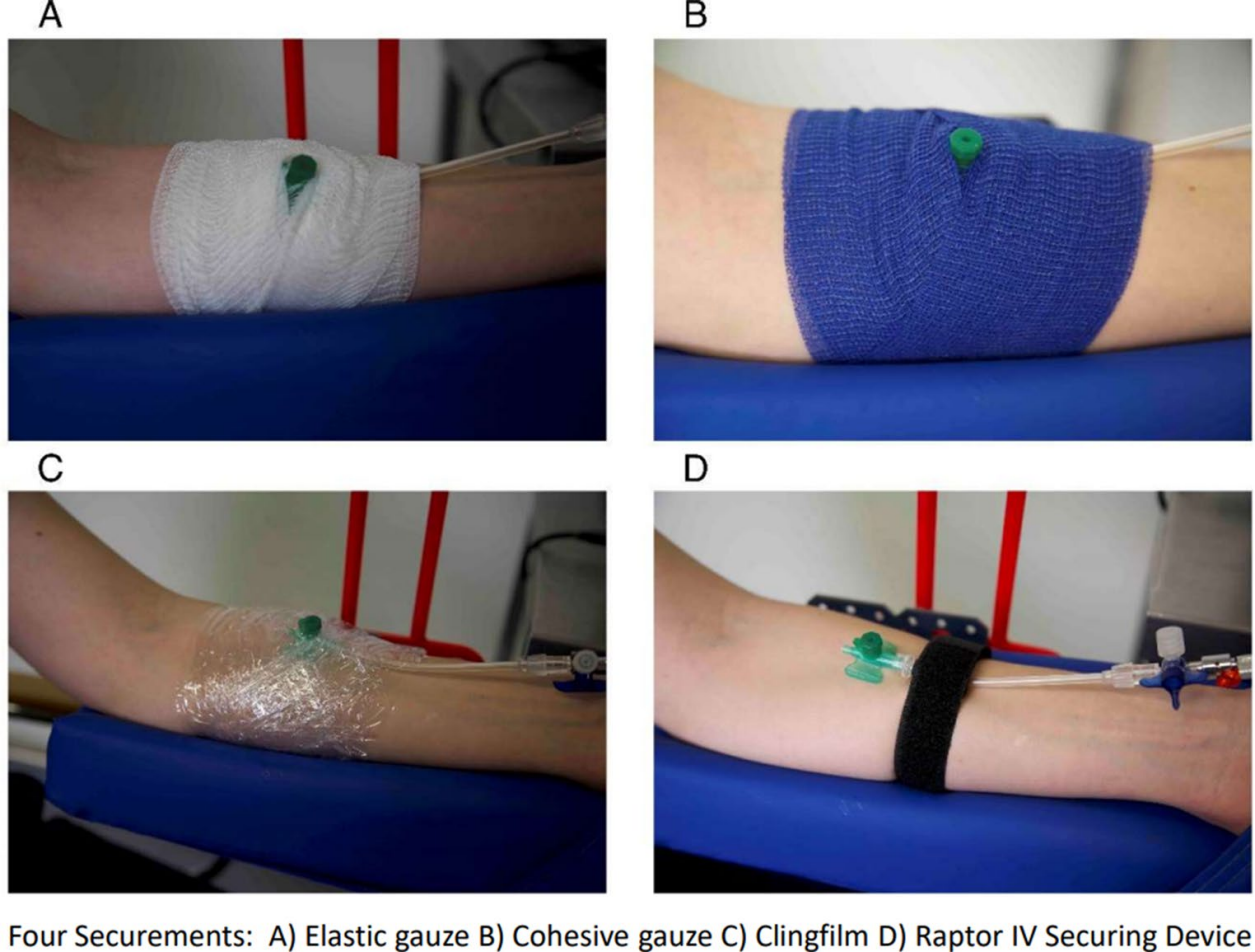
Four securements and dressings for evaluation

The following four securements and dressings were evaluated (Fig. 1):

A) Elastic Gauze (“Leukoplast Elastomull hospital“, 6 cm width).
B) Cohesive Gauze/Coban Wrap (“Leukoplast Elastomull haft color hospital“, 6 cm width).
C) Plastic wrap, “clingfilm”, (10 cm width)
D) Raptor IV Securing Device “Velcro Bandage”.

The intravascular part of an 18 gauge i.v. cannula with injection port (Vasofix Safety, B Braun, Melsungen, Germany) was truncated and the remaining part was fixated on the moistened (artificial sweat; DIN EN ISO 105-E04, Synthetic Urine e.K. – Eberdingen-Nussdorf, Germany) forearm via one of the above mentioned securements. All securements were attached by the same investigator (J.V.) in the same manner, wrapping around three times proximal and distal to the injection port of the i.v. cannula.

The sequence of securement methods was randomized prior to the study using a list randomizer (www.random.org). After attaching the randomized securement method, the pulling force on the securement was continuously increased until the truncated PIVC had moved 3.5 cm in the direction of the force or until the connection tube was torn apart. The distance was tracked visually, aided by a self-designed, 3-D-printed visual aid.

### Measurements

#### Force measurement

**Fig. 2:**
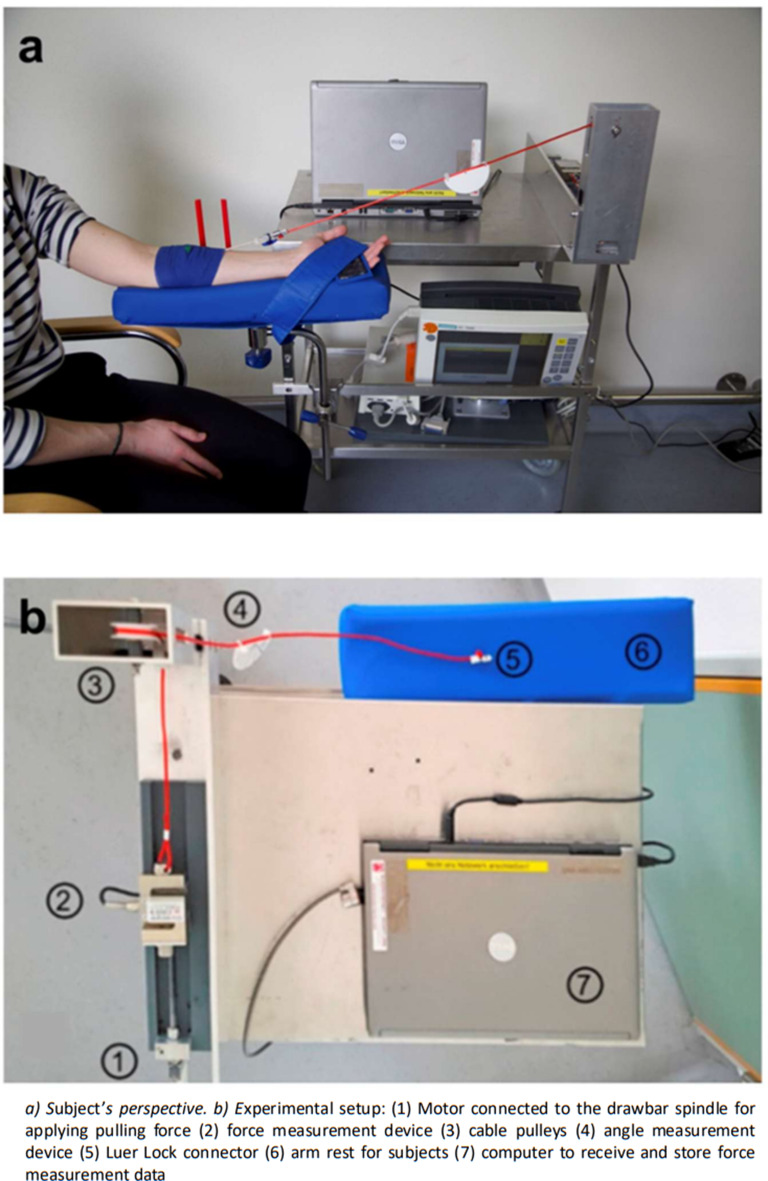
Force measurement experimental setup

A Luer lock adapter was attached to the Luer lock plug of a three-way stopcock with connection tubing, (Discofix C-3,1.2 × 2.2, B.Braun, Melsungen, Germany) attached to the truncated 18 gauge i.v. catheter. The free end of the Luer lock adapter was attached to a cord that was arranged at an angle of 30° to the horizontal. The cord was then passed through two deflection rollers and attached to a force transducer (PSD-S1 Load cell, Zhengzhou Pushton Electronic Instruments Co.,Ltd, Zhengzhou, China). The force transducer was connected to a drawbar spindle. To increase tension on the catheter securement the drawbar spindle was constantly turned by a direct current motor with constant rotational speed (Fig. 2). The peak force occurring before dislodgement was determined from continuous force recordings.

#### Small movements

To evaluate the resistance of each securement method against small movements, the steepness of the force curves between a measured force of 0.75 N and 10 N was calculated. A steeper force curve indicates a higher force necessary to cause movement of the PIVC. Movement of the PIVC in the direction of the sensor caused the force curve to flatten.

### Retest reliability

To determine the level of precision of the force sensor, the dislodgement force of the securements were measured repetitively under identical conditions (same subject) in a previous study [10]. Each dressing was allocated to one of the authors and was dislodged 20 times in a row from an identical position (antecubital region). The retest-reliability of all tested securements was comparable and did not show systematic trends.

### Outcomes

The primary endpoint was the maximum dislodgment force. The secondary endpoint was the gradient of the force curve between 0.75 N and 10 N, representing the resistance against small movements. A steep increase in force over time during the application of tension represents little or no micromovement, whereas a slow increase in force over time represents a large micromovement. Costs were calculated for each securement method, based on 2024 prices in Euro (1 EUR = 1.09 US$). Costs for staff time to apply securements were not considered.

### Data processing and statistical analysis

Raw data were collected and analyzed via software self-written in LabVIEW 7.1 (National Instruments, Austin TX) and MATLAB (MathWorks, Natick, MA, USA) and resulting force values were transferred to R (R Foundation for Statistical Computing, Vienna, Austria) for statistical processing. The minimum number of subjects was determined by a sample size calculation carried out ahead of the study. The number of cases to achieve a desired power of 95% at an alpha error of 5% was determined to be 175. The basis for the sample size calculation were preliminary test runs with the authors as subjects for the evaluation of the test set-up. Continuous variables were examined for normal distribution using the Shapiro-Wilk-Test and for variance of homogeneity using Levene’s test. Since we were dealing with repeated measurements depending on whether or not requirements were met, either Anova with repeated measurements or Friedman’s tests were carried out to analyze the data.

## Results

### Characteristics of study subjects

180 subjects gave written informed consent to participate in the study. Five of the series of measurements had to be abandoned either due to the subjects´ arm not being fixated properly or due to the fixation method not being used properly. Other statistical outliers were accepted as valid as there were no anomalies recorded by the author. The subjects’ characteristics are shown in Table 1.

**Table 1:** Subjects’ characteristics

Characteristic	*n* = 175
age [years] median [range]	30 [18–67]
sex [female/male/diverse]	110/65/0

### Main results

#### Force measurements

**Fig. 3:**
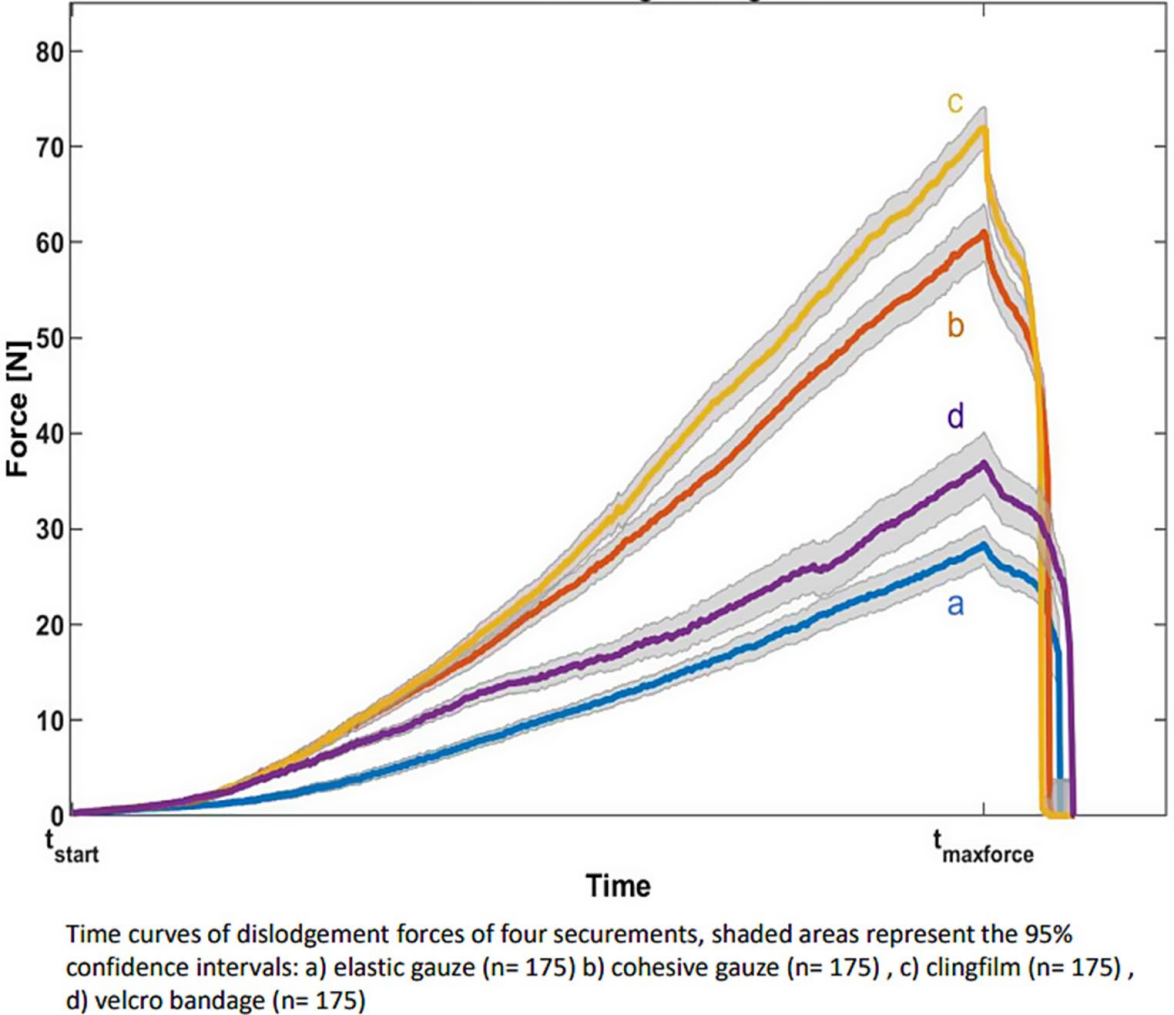
Forcecurve during dislodgement

Force measurements showed that the force increased over time until the PIVC was moved by 3.5 cm (Fig. 3).

**Fig. 4:**
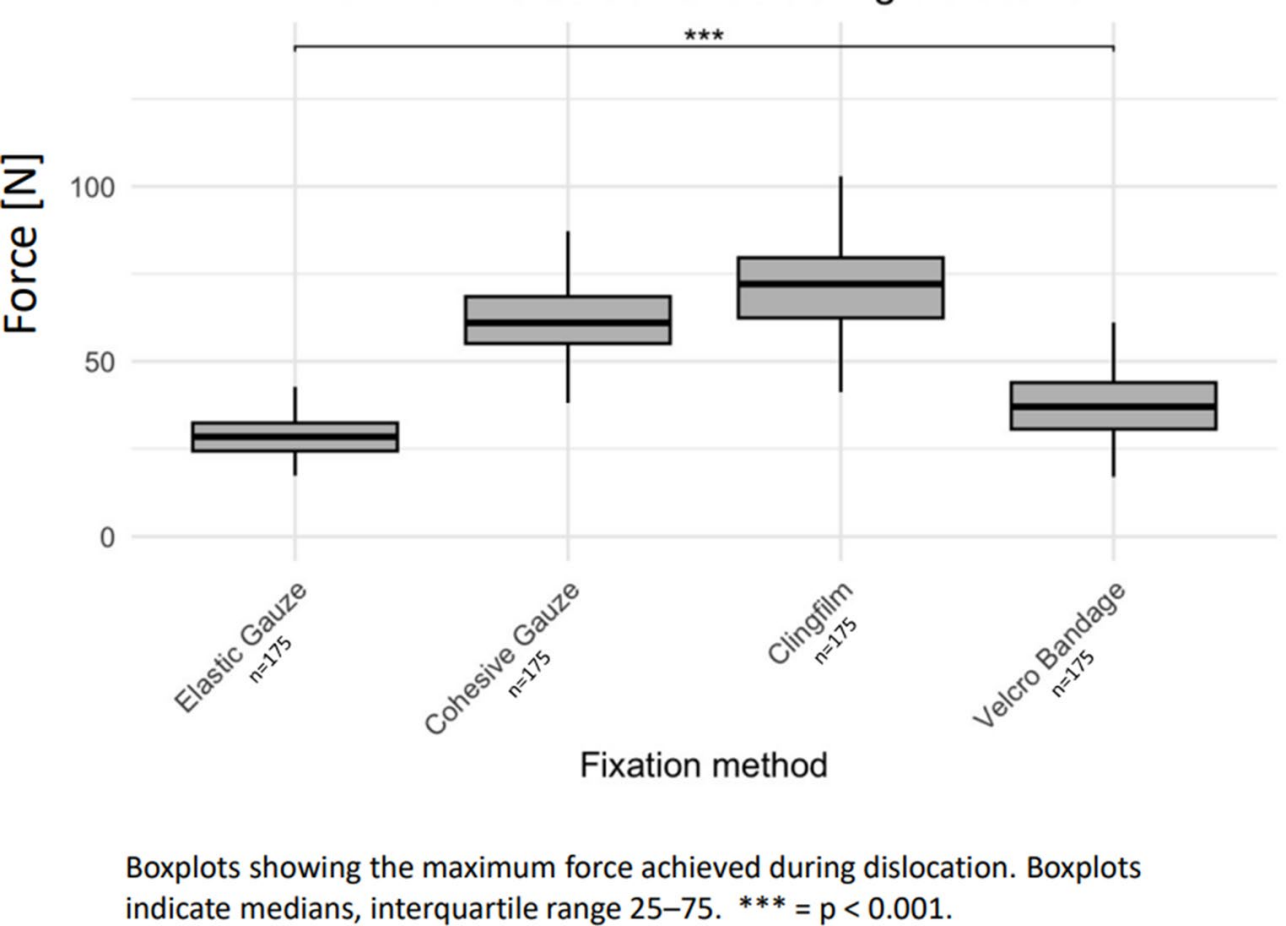
Maximum force achieved during dislocation

The highest forces before dislodgement were achieved by securement via clingfilm (72.1 N median) and cohesive gauze (61.0 N median). Elastic gauze showed the weakest results (28.4 N median) (Fig. 4).

Force gradients were highest when securing the PIVC via the velcro bandage. Cohesive gauze and clingfilm showed comparable results, leaving behind the elastic gauze.

#### Sex differences

There were no significant differences in dislodgement forces between women and men.

#### Costs

The velcro bandage (Raptor IV Securing Device) was 25 times more expensive than the elastic bandage whereas clingfilm was the most cost-effective fixation method (Table 2).

**Table 2:** Costs (€) of the four securement methods

Fixation	Manufacturer	Cost per patient
Elastic Gauze	BSN Medical GmbH	12ct
Cohesive Gauze	BSN Medical GmbH	60ct
Clingfilm	Europack24	4ct
Raptor IV Securing Device	North American Rescue	316ct

Costs of the securement method in EURO (€); ct = Eurocent

## Discussion

To our knowledge, this is the first in vivo study to provide objective force measurements regarding the effectiveness of different securement methods for PIVC in prehospital settings.

The most important finding of our study is that both clingfilm and cohesive bandages showed the best overall results as securements used in a setting where standard intra-hospital securements fail.

We looked at the maximum force that was generated when applying a longitudinal, pulling force to a secured PIVC at an angle of 30° to the horizontal. A recent in vitro study showed significantly less resistance to pull-out forces compared to our data [11]. This could be caused by the fact that the securements do not adhere to artificial skin to the same extent. An Israeli study has already compared different methods used in military medicine but did not report on force measurements. The authors also describe clingfilm as advantageous in terms of simplicity, speed of fixation and resistance to traction [9].

Cling film and cohesive bandages showed in our study the greatest resistance to high pulling forces. Both showed also comparable ability to resist small forces. It remains to be discussed whether there is a difference in comfort between the two methods. Poor comfort leads to poor patient compliance and puts the PIVC at risk.

Clingfilm has a distinct advantage over cohesive bandage in that it allows a clear, direct view of the PIVC insertion site, as it is transparent. Moreover, clingfilm is much cheaper than cohesive dressings.

However, the comparison of the costs cannot be considered to be definitive, as the clingfilm used in our study was a product designed for packaging purposes and was not an official health care product. The Raptor™ IV Securing Device velcro system, by far the most expensive product, showed weak resistance to high pulling forces, but remarkable strength as the strongest device in resisting small movements.

Small movements of the catheter inside the vein have been shown to cause irritation and a higher risk of phlebitis and therefore PIVC failure [5]. Therefore, not only the complete dislodgement but also small movements should be avoided. Elastic Bandages showed by far the lowest resistance to small forces and were unable to provide much resistance to pulling force. They also obscure the insertion site and can potentially give a false sense of security. In prehospital emergency care, vascular access is of paramount importance. Therefore, securement of PIVC is critical, and insufficient fixation of the PIVC is a major contributing factor to failure. Patient movement, transport over difficult terrain, and changes in position can all exert pulling forces on the attached i.v. lines, ultimately causing the PIVC to become dislodged and unintentionally removed.

So far there are no guidelines addressing the optimal securement method for prehospital PIVC. In our opinion, however, these are necessary.

## Limitations

This single-center, randomized study design was inevitably not blinded. The securements were only in place for a short period of time until the pulling force was applied. Therefore, the effects of movement, repetitive pulling and wearing were not taken into account. There are many different PIVC devices, some of which do not have securement wings, unlike the devices we used. The dislodgement forces may differ depending on which PIVC device is used, which limits the generalisability of the results.

We have only investigated longitudinal traction forces at an angle of 30° to the horizontal, although the various potential directions of forces make it difficult to study this issue further. However, a recent in vitro study showed less resistance to pull-out force when the angle was 30° compared to 0° [11]. We did not place the i.v. catheters into the veins of the subjects. However, we think that an additional intravascular part of the catheter would not have changed our results by adding a relevant resistance to the pulling forces. The methods we used do not provide a sterile cover for the PIVC insertion site, thereby increasing the risk of infection. A transparent dressing for cleanliness may also provide a wider surface area for a wrap to help hold down. For Patients at higher risk of PIVC dislodgement, the fixation methods we used are usually employed alongside standard PIVC dressings. It could be argued that this combination yields different results with regard to dislodgement forces. The Raptor IV Securing Device is unlikely to be used alone to secure a PIVC, as it only secures the IV tubing, not the PIVC itself. Using it alongside adhesive dressings would be more reasonable and could show different results with regard to dislodgement forces.

Dislodgement of the PIVC is likely to occur well before a movement of 3.5 cm. 3.5 cm corresponds to the length of the intravascular part of a PIVC, meaning that a movement of 3.5 cm is a point of definitive dislodgement.

We have not evaluated the effect of a circular securement on IV line flow [12]. The strength of circular securement methods depends on the force with which they are applied. This in turn is limited by vascular constriction. To counteract this effect, each test was performed by the same person in as similar circumstances as possible.

## Conclusions

In situations where a PIVC is at risk for dislodgement (i.e. wet skin, confused patients, rough terrain) and normal bandage securements are not applicable, either clingfilm or cohesive bandages should be the first choices for fixation. These methods should also be the first choice when looking for secondary movement to a standard securement bandage. Elastic bandages performed by far weakest in our tests and should not be used to secure PIVCs. At worst they also hinder visual inspection of the insertion site and may provide a false sense of security.

Applying the strongest securement dressing to prehospital PIVC may reduce the failure rate and thus improve patient care and safety, and is also cost-effective. We therefore recommend the use of either cling film or cohesive bandages as a securement for prehospital applied PIVC.

## Data Availability

The datasets used and/or analyzed during the current study are available from the corresponding author on reasonable request.

## References

[1] Golling E, van de Mortel T, Barr N, Zimmerman PA. Pre-hospital peripheral intravenous catheter insertion practice: an integrative review. Australasian Emerg Care. 2023;26(2):105–12. 10.1016/j.auec.2022.08.006. 10.1016/j.auec.2022.08.006 36117094

[2] Marsh N, Webster J, Larson E, Cooke M, Mihala G, Rickard CM. Observational study of peripheral intravenous catheter outcomes in adult hospitalized patients: a multivariable analysis of peripheral intravenous catheter failure. J Hosp Med. 2018; 13(2): 83–9. 10.12788/jhm.2867. PMID: 29073316. 10.12788/jhm.2867 29073316

[3] Moureau N. Impact and safety associated with accidental dislodgement of vascular access devices: a survey of professions, settings, and devices. J Associ Vasc Access. 2018;23(4):203–15. 10.1016/j.java.2018.07.002.

[4] Macklin D, Phlebitis. A painful complication of peripheral IV catheterization that may be prevented. AJN Am J Nurs. 2003;103(2).

[5] Corley A, Ullman AJ, Mihala G, Ray-Barruel G, Alexandrou E, Rickard CM. Peripheral intravenous catheter dressing and securement practice is associated with site complications and suboptimal dressing integrity: a secondary analysis of 40,637 catheters. Int J Nurs Stud. 2019;100:103409. 10.1016/j.ijnurstu.2019.103409. 31629208 10.1016/j.ijnurstu.2019.103409

[6] Marsh N, Mihala G, Ray-Barruel G, Webster J, Wallis MC, Rickard CM. Inter-rater agreement on PIVC-associated phlebitis signs, symptoms and scales. J Eval Clin Pract. 2015;21(5): 893–9. 10.1111/jep.12396. PMID: 26183837. 10.1111/jep.12396 26183837

[7] Rickard CM, Marsh N, Webster J, et al. Dressings and securements for the prevention of peripheral intravenous catheter failure in adults (SAVE): a pragmatic, randomised controlled, superiority trial. Lancet. 2018;392(10145):419–30. 10.1016/S0140-67361831380-1. PMID: 30057103. 10.1016/S0140-6736(18)31380-1 30057103

[8] Marsh N, Webster J, Mihala G, Rickard CM. Devices and dressings to secure peripheral venous catheters: a cochrane systematic review and meta-analysis. Int J Nurs Stud. 2017;67:12–9. 10.1016/j.ijnurstu.2016.11.007. PMID: 27889585. 10.1016/j.ijnurstu.2016.11.007 27889585

[9] Fried E, Shochat T, Har-even Y, Cohen Y. Evaluation of intravenous cannula and administration set fixation methods. Mil Med. 2005;170(11):931–4. 10.7205/MILMED.170.11.931. 16450820 10.7205/milmed.170.11.931

[10] Schmutz A, Menz L, Schumann S, Heinrich S. Dislodgement forces and cost effectiveness of dressings and securement for peripheral intravenous catheters: a randomized controlled trial. J Clin Med. 2020. 10.3390/jcm9103192. 33019691 10.3390/jcm9103192 PMC7601033

[11] Benjamin R, Mackie, et al. Securing cannulas in combat: an in vitro study of three intravenous securement techniques. J High Threat Austere Med. 2023;5(1). 10.33235/JHTAM.5.1.3-9.

[12] Morgan TR. Evaluation of fluid bolus administration rates using ruggedized field intravenous systems. Wilderness Environ Med. 2014;25(2):204–9. 10.1016/j.wem.2013.12.026. PMID: 24631229. 10.1016/j.wem.2013.12.026 24631229

